# Prevalence of Anxiety Among Medical, Dental, and Nursing Students in Selected Balkan Countries: A Systematic Review and Meta-Analysis

**DOI:** 10.3390/jcm15218566

**Published:** 2026-11-03

**Authors:** Cătălin Pleșea-Condratovici, Constantin-Marinel Vlase, Ionuț-Silviu Vasile, Aritina-Elvira Moroșanu, Manuela Arbune, Petruț-Ștefan Șerban, Răzvan Cosmin Pană

**Affiliations:** 1 Morphological and Functional Sciences Department, Faculty of Medicine and Pharmacy, “Dunărea de Jos” University of Galaţi, 800008 Galati, Romania; catalin.plesea@ugal.ro; 2 Clinical Medical Department, Faculty of Medicine and Pharmacy, “Dunărea de Jos” University of Galaţi, 800008 Galati, Romania; constantin.vlase@ugal.ro (C.-M.V.); manuela.arbune@ugal.ro (M.A.); 3 “Dr. Aristide Serfioti” Military Emergency Hospital, 800008 Galati, Romania; 4 Discipline of Endocrinology, Department 5, University of Medicine and Pharmacy of Craiova, 200349 Craiova, Romania; 5 Pediatrics Department, University of Medicine and Pharmacy of Craiova, 200349 Craiova, Romania; 6 Faculty of Medicine and Pharmacy, “Dunărea de Jos” University of Galaţi, 800008 Galati, Romania; serban.stefan.univ@gmail.com; 7 2nd Department, Faculty of Nursing and Midwifery, University of Medicine and Pharmacy of Craiova, 200349 Craiova, Romania; razvancosminpana@yahoo.com

**Keywords:** anxiety, medical students, dental students, nursing students, Balkans, meta-analysis, systematic review, mental health, prevalence, health science education

## Abstract

**Background:** Anxiety is common among health science students, but the Balkan region is underrepresented in student mental health research and no regional synthesis exists. This systematic review and meta-analysis estimated the pooled prevalence of anxiety among medical, dental and nursing students in selected Balkan countries. **Methods:** Following PRISMA 2020 and a PROSPERO registration (CRD420261332221), PubMed, Scopus, and Web of Science were searched for English-language observational studies in eleven Balkan countries that assessed anxiety with a validated instrument. Two reviewers independently screened studies, extracted data and appraised quality (JBI). Prevalence was pooled with a DerSimonian-Laird random-effects model after Freeman-Tukey transformation, with prediction intervals and exploratory subgroup and sensitivity analyses. **Results:** Nine cross-sectional studies (*N* = 5177) from Croatia, Romania, Greece, Kosovo and Serbia were included. The pooled prevalence of anxiety at or above moderate severity was 27.5% (95% CI: 20.2–35.5%), with very high heterogeneity (*I*^2^ = 97.1%; 95% prediction interval 5.3–58.6%). Leave-one-out analyses (26.0–29.4%) and alternative estimators (26.9–27.5%) gave similar estimates. Subgroup analyses were exploratory. **Conclusions:** The pooled prevalence is an average across heterogeneous studies, not a regional benchmark. The evidence is limited to English-language studies from five of eleven countries, and the findings are preliminary. Harmonised primary research is needed.

## 1. Introduction

Anxiety disorders represent one of the most prevalent and burdensome categories of mental illness worldwide, affecting an estimated 301 million people globally and constituting a leading cause of disability-adjusted life years across all age groups [1,2]. According to the Global Burden of Disease 2021 study, the global prevalence of anxiety disorders reached approximately 359.2 million cases, accounting for 9.1% of all diseases and 63.1% of all mental health disorders, with projections indicating that case numbers could surpass 515 million by 2040 if current trajectories continue [3]. Although anxiety disorders are highly treatable, they remain widely underdiagnosed and undertreated, particularly in lower-resource healthcare systems where access to mental health services is limited [4,5,6]. Within the European context, adequate treatment coverage for anxiety disorders varies dramatically—from approximately 7% in Bulgaria to 47% in Sweden—underscoring profound inequities in mental health care provision across the continent [4].

The figures above refer to clinically diagnosed anxiety disorders, whereas most epidemiological studies in students, including those synthesised in the present review, measure self-reported anxiety symptoms with screening instruments such as the GAD-7, DASS-21 and HADS [7,8,9]. These instruments identify probable cases above a chosen severity threshold rather than confirm a diagnosis, and their accuracy against diagnostic interview is imperfect [10]; throughout this manuscript, ‘anxiety’ therefore denotes clinically relevant anxiety symptoms, not diagnosed anxiety disorders. University students represent a particularly vulnerable population for the emergence and escalation of anxiety symptoms, as the transition into higher education coincides with significant developmental, social, and academic challenges [11,12]. Global meta-analytic evidence consistently reports a pooled prevalence of anxiety symptoms of approximately 30–45% among undergraduate students worldwide, substantially exceeding rates observed in the general adult population [13,14]. Among health science students—including those enrolled in medicine, dentistry, and nursing programmes—this burden appears even more pronounced. A systematic review and meta-analysis by Ahmed et al. (2023) examining 83 studies involving over 100,000 undergraduate students reported a pooled prevalence of non-specific anxiety of 39.7% (95% CI: 35.7–43.6%), and 37.4% (95% CI: 30.8–44.1%) in samples of medical students only [14]. This figure reflects the particular psychosocial demands imposed by health-professional training environments and highlights the need for field-specific research and interventions.

The elevated prevalence of anxiety in medical students has been linked to a convergence of stressors that are largely unique to health professions education. The training environment demands extreme academic dedication, with heavy workloads, inconsistent sleep schedules, and a marked imbalance between study and leisure [15,16]. Students are further exposed to emotionally taxing experiences such as contact with patient suffering, the fear of clinical errors, high-stakes examinations, and the competitive atmosphere of medical institutions [16,17]. Although the three programmes differ in curriculum, clinical setting and professional role, they share clinical training, patient contact and high-stakes assessment [16,17,18,19,20,21], which motivates examining them together. We do not assume, however, that they share a single true prevalence: a random-effects model estimates the mean of a distribution of study-specific prevalences [22], and discipline is examined as a source of heterogeneity in exploratory subgroup analyses. For nursing students in particular, the emotional intensity of clinical exposure—including confrontations with suffering, death, and critical illness—constitutes a recurring source of distress that distinguishes their training from most other academic programmes [18,19]. For dental students, anxiety has been associated with the fear of causing harm to patients, procedural performance pressure, and the interruption of clinical training, as was acutely demonstrated during the COVID-19 pandemic [20,21]. Sex, year of study, financial hardship, perceived social support, and personality traits such as neuroticism have been repeatedly identified as significant correlates of anxiety across health professions student populations [16,17].

The consequences of untreated or undertreated anxiety in medical students extend well beyond individual wellbeing. Longitudinal evidence demonstrates that elevated anxiety is associated with impaired attentional processes, working memory deficits, and reduced cognitive flexibility—all mechanisms critical to both academic learning and safe clinical practice [23,24]. Anxiety has been shown to predict lower grade point averages, increased absenteeism, greater procrastination, reduced academic engagement, and elevated risk of dropout [23,24]. Beyond the academic domain, anxiety is a significant predictor of burnout, clinical misconduct, and, in the most severe cases, suicidal ideation among medical students [16,25]. The downstream implications of student mental health are therefore not limited to individual trajectories but extend to future healthcare delivery and patient safety.

While the global evidence base on anxiety in medical students has grown considerably over the past decade, substantial regional gaps persist. The Balkans—operationally defined here as the eleven Southeastern European countries Albania, Bosnia and Herzegovina, Bulgaria, Croatia, Greece, Kosovo, Montenegro, North Macedonia, Romania, Serbia and Slovenia, which are the countries searched and eligible in this review (Section 2.2)—is not a homogeneous setting: educational systems, economic conditions and mental health services differ considerably between countries [4,6]. The Balkans are best understood as a historical and cultural region rather than a precisely bounded political unit. For centuries, most of these countries belonged to, or bordered, the Ottoman and Habsburg empires, and in the twentieth century many of them went through the formation of new states, political and economic transition and, in some cases, armed conflict. Societies in the region are also often described as close-knit and family-centred. We use this shared historical and social background as a rationale for examining the region together in a first synthesis, without assuming that students in all countries are exposed to the same conditions. These countries nevertheless share features relevant to student mental health, including comparatively low treatment coverage for anxiety disorders, limited investment in community mental health services and persistent stigma [4,6,26], which makes the region a meaningful unit for a first synthesis; country-level differences are examined in exploratory subgroup analyses. It is a region where mental health research among student populations remains comparatively sparse and methodologically inconsistent [6,27]. A meta-analysis synthesising evidence from Eastern Europe during the COVID-19 pandemic reported a pooled anxiety prevalence of 31% among students in the region [27], while a systematic review of Croatian student populations documented anxiety symptom rates as high as 76.5% (GAD-7, any severity) among Croatian health science students in one reviewed study [28]. Despite these concerning figures, the Balkans region has historically been underrepresented in international mental health surveillance, evidenced by a dearth in the dedicated literature and a lack of region-specific syntheses [6,27,29]. Furthermore, recent changes in healthcare systems, underfunding of community mental health resources, and persistent stigma surrounding psychiatric illness create a unique context in which evidence-based mental health practice has been slow to develop [6,26].

To our knowledge, no systematic review and meta-analysis has specifically quantified the pooled prevalence of anxiety among medical, dental, and nursing students in selected Balkan countries. This was checked by searching PubMed, Scopus, Web of Science and PROSPERO on 30 September 2026 for reviews combining student, anxiety and regional terms (Supplementary Table S1); the closest existing syntheses concern students in Eastern Europe during the COVID-19 pandemic [27] and Croatian students during the pandemic [28]. The absence of such a regional synthesis leaves policymakers, academic institutions, and mental health professionals without the aggregated evidence necessary to design targeted interventions, allocate resources appropriately, and interpret local institutional findings in a regional context. This gap is particularly significant given that anxiety, although comparably prevalent and debilitating to depression, has historically received less systematic attention in the academic mental health literature, even at the global level [14,15]. The present study therefore aims to address this critical gap through a systematic review and meta-analysis of published studies reporting the prevalence of anxiety among medical, dental, and nursing students across Balkan countries. Specifically, this study seeks to: (1) estimate the pooled prevalence of anxiety among health science students in selected Balkan countries through a random-effects meta-analysis; (2) explore heterogeneity through exploratory subgroup analyses by country, health science discipline, assessment instrument, and a study-level meta-regression on the proportion of female participants; and (3) provide a synthesis of the available regional evidence to inform future institutional and national studies. By synthesising the available evidence, this study aims to contribute to a more robust and contextually informed understanding of student mental health in Southeastern Europe and to support the development of preventive strategies within Balkan medical education systems.

## 2. Materials and Methods

This systematic review and meta-analysis was conducted and reported in accordance with the Preferred Reporting Items for Systematic Reviews and Meta-Analyses (PRISMA) 2020 guidelines [30] (the completed checklist is provided in Supplementary Materials, Table S4) and was prospectively registered in the PROSPERO international register of systematic reviews (registration number: CRD420261332221).

### 2.1. Search Strategy

A systematic literature search was conducted across three electronic databases: PubMed, Scopus, and Web of Science. No date restriction was applied in the database queries. Records published after 31 March 2026 were excluded manually after export, before eligibility assessment. This cut-off is the date of the first search and was therefore defined before the re-run; it was kept fixed so that the review period remained the one defined at the first search, and the purpose of the re-run was to capture records published up to that date but indexed afterwards, not to extend the review period. Eight records dated after 31 March 2026 were excluded under this rule. Searches were run on 31 March 2026 and re-run on 29 September 2026; both runs also applied an open-access filter. After peer review, this open-access restriction was removed (Section 2.7) and the three searches were repeated on 12 October 2026 with the same search strings, without the open-access filter. Search strategies were tailored to each database’s syntax and combined controlled vocabulary and free-text terms relating to the student population, the outcome of interest (anxiety), and the target geographic region. The population, outcome and geographic concept blocks were kept conceptually equivalent across the three databases (medical, nursing, dental, dentistry and health science students; anxiety; the eleven countries listed in Section 2.2). The full search strings for each database are provided in Appendix A.

### 2.2. Eligibility Criteria

Studies were included if they met all of the following criteria: (1) the study population consisted of students enrolled in health science programmes, namely medicine, dentistry or nursing; samples that also included students of other health-profession programmes (for example physiotherapy or radiological technology) were eligible when medicine, dentistry or nursing students made up the majority of the sample and the results for the other programmes could not be separated; (2) the study was conducted in one of the following Balkan countries: Albania, Bosnia and Herzegovina, Bulgaria, Croatia, Greece, Kosovo, Montenegro, North Macedonia, Romania, Serbia, or Slovenia; (3) the prevalence of anxiety was assessed using a validated psychometric instrument (e.g., the Generalized Anxiety Disorder 7-item scale [GAD-7], the Beck Anxiety Inventory [BAI]); and (4) the study design was a primary observational study (cross-sectional or cohort). Studies were excluded if they: reported no extractable prevalence data; used non-validated or non-standardised anxiety assessment tools; were review articles, meta-analyses, editorials, case reports, or qualitative studies; or were not available in English. The English-language restriction was applied from the first search (31 March 2026), before any record was screened, as a feasibility measure, so that every full text could be assessed and the extracted data verified by every reviewer. It is a recognised limitation, because relevant studies published in regional languages may have been missed (Section 4.4 and Section 4.6). An initial restriction to open-access publications was removed after peer review: open-access status is unrelated to study quality or population and can introduce availability bias, so the searches were repeated without it and all additional records were screened under the same criteria (Section 2.1, Section 2.3 and Section 2.7). Definitions of the Balkan region vary in the literature (for example, Romania, Slovenia and Greece are included in some definitions and not in others); we therefore adopted an inclusive operational definition of eleven Southeastern European countries to avoid omitting potentially eligible studies, and results are reported by country. Throughout this manuscript, ‘health science students’ is used as an umbrella term for these populations. It does not imply a homogeneous population, and discipline-specific prevalence is not claimed unless explicitly stated; ‘medical students’ refers only to samples of students of medicine. Studies with data collected between 11 March 2020 and 31 December 2022 were excluded because pandemic restrictions (lockdowns, remote teaching, social isolation) are an external factor acting on student anxiety that may inflate prevalence estimates, and pooling such periods with non-pandemic periods would make the pooled estimate difficult to interpret. The end date is a pragmatic calendar boundary approximating the lifting of restrictions during 2022 (the WHO declared the end of the COVID-19 public health emergency of international concern on 5 May 2023); we acknowledge that the transition was gradual and country-specific and that no single date is uniquely justified. Data collected from January 2023 onwards were treated as post-restriction data. For two included studies (Hoxha et al. [31] and Ilić Živojinović et al. [32]), the data-collection period was reported only as an academic year or semester (2022/2023 and winter semester 2022, respectively), so overlap with the pandemic exclusion window (11 March 2020 to 31 December 2022) could not be ruled out with certainty; both were retained under this rule, and their influence was examined in a sensitivity analysis (Section 3.5). The data-collection period of Milić et al. (2019) [33] was not reported, but the article was received on 7 September 2018, so its data predate the pandemic window. The exclusion was applied only where data collection was documented to fall within the window, because excluding studies on the basis of an assumed overlap would have been equally arbitrary; excluding both studies gave a higher pooled prevalence (29.7%, 95% CI: 21.4–38.8%) than the primary estimate, so their retention did not inflate it.

### 2.3. Study Selection

**Figure 1:**
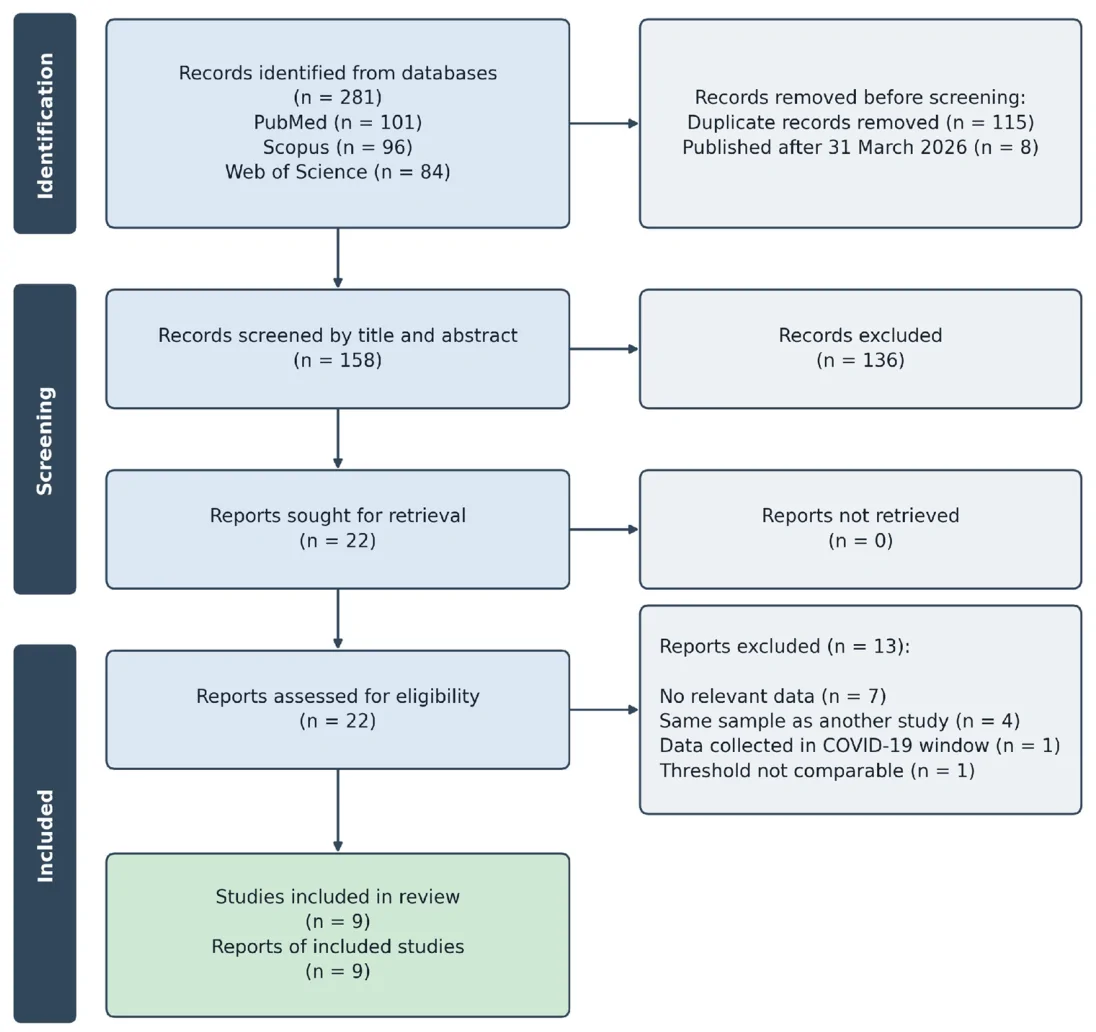
PRISMA 2020 flow diagram.

All retrieved records were imported into Rayyan (Rayyan Systems Inc., Cambridge, MA, USA) [34] for duplicate removal and screening. Following de-duplication, two authors (A.-E.M. and M.A.) independently screened all titles and abstracts for eligibility. Full texts were subsequently retrieved and assessed independently by the same two reviewers for all records passing the initial screen. Agreement between the two reviewers was complete (Cohen’s *κ* = 1.00) at both stages, so no third-party arbitration was required. Of the 281 records identified, 115 duplicates were removed (48 records were indexed in all three databases, 19 in two and 99 in one), leaving 166 unique records; 8 of these were removed because they were published after 31 March 2026. The remaining 158 records were screened by title and abstract, 22 full texts were assessed, and 9 studies were included. Of the 166 unique records, 44 had not been retrieved by the earlier open-access searches; one of them (Milić et al., 2019 [33] met all eligibility criteria and was included, and one report was excluded after full-text assessment (Supplementary Table S2). Reasons for full-text exclusion are shown in Figure 1 and Supplementary Table S2. When several reports drew on the same population, one report was retained: the one with the largest sample and an extractable prevalence at the required threshold. Milić et al. [33] (2019; Osijek, data collected in 2018 or earlier) and Vidović et al. [35] (2025; January–February 2025) concern different student cohorts and were both retained (Supplementary Table S3). The study selection process is illustrated in the PRISMA 2020 flow diagram (Figure 1).

### 2.4. Data Extraction

Data were extracted independently by two authors (C.P.-C. and R.C.P.) using a standardised extraction form. The following variables were recorded for each included study: title, year of publication, year of data collection, journal, DOI, country, study design, type of student population, sample size, number of anxiety cases, anxiety prevalence, anxiety cut-off applied, anxiety classification categories reported, assessment instrument used, mean age and standard deviation of participants, and percentage of female participants.

### 2.5. Quality Assessment

The methodological quality of all included studies was assessed independently by two reviewers (C.-M.V. and R.C.P.) using the Joanna Briggs Institute (JBI) Critical Appraisal Checklist for Studies Reporting Prevalence Data [36]. This tool evaluates studies across nine domains, including the appropriateness of the sampling frame, the adequacy of the sampling method, the sample size, the description of study subjects and setting, data analysis, the validity and reliability of the measurement instrument, and the appropriateness of statistical analysis. Each criterion was rated as “Yes”, “No”, “Unclear”, or “Not Applicable”. Overall risk of bias was categorised as low (≥7 criteria met), moderate (4–6 criteria met), or high (≤3 criteria met); these thresholds are author-defined, as the JBI tool does not specify a total-score cut-off, and the summary category should be read together with the domain-level appraisal (Section 3.2), which is the primary basis for judging the risk of bias of each study. Disagreements were to be resolved by consensus; none arose.

### 2.6. Statistical Analysis

All statistical analyses were performed using Python (version 3.10; Python Software Foundation, Wilmington, DE, USA) with the numpy (version 2.2.6) and scipy (version 1.15.3) libraries [37,38]. The primary outcome was the pooled prevalence of anxiety among health science students in selected Balkan countries. Raw prevalence proportions from each included study were transformed using the Freeman-Tukey double arcsine transformation prior to pooling, to stabilise variances across studies with proportions near 0 or 1 [39]. Back-transformation was applied to all pooled estimates for reporting. The prevalence in each study is the proportion of participants above the cut-off applied in that study: moderate severity for the DASS and GAD-7 and ≥8 for the HADS-A. These operational definitions are not equivalent (the HADS-A cut-off of ≥8 identifies borderline or abnormal scores and is lower than a moderate-severity threshold), so the pooled estimate is interpreted as a combination of study-specific screening definitions rather than a single severity level; the HADS-A study was examined in sensitivity analyses.

Pooled prevalence was estimated with a DerSimonian-Laird random-effects model [40], because the included studies were expected to differ in population, instrument and setting. Between-study heterogeneity was assessed with Cochran’s *Q*, the *I*^2^ statistic and the between-study variance τ^2^ [22]. Pooled estimates are reported with 95% confidence intervals and with 95% prediction intervals (t distribution with *k* − 2 degrees of freedom), which describe the range in which the true prevalence of a new study or setting is expected to fall. In the forest plot, the prevalence of each study is shown as the raw proportion with a Wilson 95% confidence interval.

To explore potential sources of heterogeneity, subgroup analyses, all exploratory, were conducted by (1) country, (2) health science discipline (medicine, nursing, dentistry, and samples combining several programmes, each combination treated as its own category), and (3) anxiety assessment instrument. Because only nine studies were available, pooled estimates were computed only for subgroups with at least three studies; subgroups with fewer studies are presented as individual study estimates without pooling, and all subgroup comparisons are considered exploratory and hypothesis-generating. Dental and nursing students were not pooled together, as they are distinct populations; only medical-student samples met the minimum of three studies required for pooling. Sex-specific anxiety prevalence was not consistently reported across the included studies, so a subgroup analysis by sex was not feasible; instead, the proportion of female participants was examined as a study-level moderator using random-effects meta-regression (REML, logit-transformed prevalence, Hartung–Knapp adjustment), an analysis that is ecological and exploratory. Sensitivity analyses were performed by restricting the pooled estimate to studies rated as low risk of bias based on the JBI appraisal. In addition, post hoc sensitivity analyses examined the relative weight of each study, the exclusion of the largest study, the exclusion of studies whose data-collection period could only be inferred from the academic year, the exclusion of studies whose samples were not restricted to medicine, dentistry or nursing programmes, and the use of a stricter HADS-A cut-off (≥11). Because of the small number of studies and the high between-study variance, the pooled estimate was also re-computed using REML and Paule–Mandel estimators of τ^2^ with the Freeman–Tukey transformation, the Hartung–Knapp adjustment with a t distribution (k−1 degrees of freedom), a logit transformation with the DerSimonian–Laird estimator, and a binomial–normal generalized linear mixed model (profile-likelihood confidence interval); these analyses were not pre-specified in the registered protocol. Because fewer than ten studies were included, funnel-plot inspection and Egger’s test [41] were not performed, as they are underpowered and unreliable with so few studies [42]; the risk of publication and availability bias was considered qualitatively (Section 4.4). Statistical significance was set at α = 0.05 (two-tailed) throughout.

### 2.7. Protocol Registration and Deviations

The protocol was registered in PROSPERO (CRD420261332221) on 5 March 2026, before the formal database searches (31 March 2026) and study selection. During the review and its revision, the following changes were made relative to the registered protocol: (i) Serbia and Slovenia, listed as Balkan countries in the Introduction, were added to the eligibility criteria and search strategies; (ii) the registered publication window (1 January 2021 to 4 March 2026) was replaced by no lower date limit and an upper limit of 31 March 2026, and studies with data collected before the COVID-19 pandemic were also considered eligible, whereas studies with data collected between 11 March 2020 and 31 December 2022 were excluded; studies not reporting the data collection period were included unless the data were described as collected during the pandemic; (iii) the anxiety threshold was set at moderate severity or above (registered: at least mild) to improve comparability across instruments; (iv) trait/state anxiety measures without a comparable moderate-severity cut-off were excluded; (v) open-access availability was added as a restriction; (vi) risk of bias was assessed with the JBI checklist for prevalence studies instead of QUADAS/QUADAS-C, which are designed for diagnostic accuracy studies; (vii) prediction intervals and sensitivity analyses were added post hoc. Consistent with current recommendations [42], the funnel plot and Egger’s test planned in the first version of the manuscript were not performed because fewer than ten studies were included. The PROSPERO record was updated on 30 September 2026 to reflect these changes. Regarding timing: the open-access and English-language filters were applied from the first search (31 March 2026), before any record was screened, and the cut-off of 31 March 2026 is the date of that search. The anxiety threshold (moderate or above) was chosen before prevalence estimates were extracted. The pandemic-period exclusion was intended from the outset of the review but was not written into the eligibility criteria of the first version of the manuscript; one study with pandemic-period data was included in the first version and removed when records were re-assessed for the revision. The addition of Serbia and Slovenia and the eligibility of pre-pandemic data were introduced during the revision, after the results of the first version were known; they corrected omissions of eligible studies in the initial search and were not made in response to the prevalence estimates, although this cannot be verified independently. The planned subgroup analysis by sex was replaced by a study-level meta-regression on the proportion of female participants, because sex-specific prevalence estimates were not reported consistently. The open-access restriction was removed after peer review, after all pooled results were known, in response to the reviewers’ comments and not to the prevalence estimates: the searches were repeated on 12 October 2026 without the open-access filter, all additional records were screened under the same criteria, and one study (Milić et al., 2019 [33] was added, which changed the pooled prevalence from 29.4% (eight studies) to 27.5%. The PROSPERO record was updated on 20 October 2026 to reflect this change.

## 3. Results

### 3.1. Characteristics of Included Studies

The nine included studies were published between 2017 and 2025 and collectively encompassed 5177 health science students across five Balkan countries: Serbia (*k* = 3), Croatia (*k* = 3), Romania (*k* = 1), Greece (*k* = 1), and Kosovo (*k* = 1). Data were collected between 2015 and 2025: in 2015 (Jovanović et al.) [43], 2018 (Nikolić et al.) [44], the winter semester of 2022 (Ilić Živojinović et al. [32]), the 2022/2023 school year (Hoxha et al. [31]), 2023 (Milić et al., 2024 [45]), 2024 (Mangoulia et al. [46]), and 2024–2025 (Plesea-Condratovici et al. [47]; Vidović et al. [35]). Milić et al. (2019) [33] did not report the data-collection period; the manuscript was received on 7 September 2018, so the data were collected in 2018 or earlier. For two of these studies (Hoxha et al. [31] and Ilić Živojinović et al. [32]), the period was reported only as an academic year or semester. Individual sample sizes ranged from 70 to 2048 students. In terms of programme, three studies included students of medicine only, one of dentistry only, one of dentistry and nursing, two of medicine and nursing (Plesea-Condratovici et al. [47]; Milić et al., 2019 [33]), one of nursing and medical technician programmes, and one a mixed health sciences sample composed mainly of nursing students (69.2%) together with students of other health professions (Milić et al., 2024 [45]). All nine studies were cross-sectional (Hoxha et al. [31] did not name the design, but data were collected at a single time point).

Anxiety was assessed using four validated psychometric instruments: the DASS-21 in four studies, the DASS-42 in one, the GAD-7 in three, and the HADS-A in one. Eight studies applied a moderate-severity cut-off (anxiety score ≥10 on the DASS subscales and the GAD-7); Plesea-Condratovici et al. [47] used the HADS-A cut-off of ≥8 (borderline abnormal or above), consistent with the validated scoring conventions of that instrument. This threshold is lower than, and not equivalent to, the moderate-severity cut-offs of the other instruments, so the prevalence reported by that study is not strictly comparable with the others; its influence was examined in sensitivity analyses (Section 3.5.1). The reported anxiety prevalence ranged from 14.7% to 41.6%, and the proportion of female participants ranged from 50.0% to 86.3%. The detailed characteristics of all included studies are presented in Table 1.

### 3.2. Results of Quality Assessment

All nine included studies were assessed for methodological quality using the JBI Critical Appraisal Checklist for Prevalence Studies (9 criteria). The appraisal is interpreted at the level of individual domains. Appropriate participant recruitment (Q2) was not met in any of the nine studies, and an adequate response rate or management of non-response (Q9) was not met in seven; the two studies that met Q9 were Ilić Živojinović et al. [32] and Milić et al. (2019) [33] (response rate 72.7%). The appropriateness of the sampling frame (Q1) was not met in two studies (Hoxha et al. [31]; Ilić Živojinović et al. [32]) and an adequate sample size (Q3) in two (Plesea-Condratovici et al. [47]; Hoxha et al. [31]). These limitations are characteristic of convenience-sampled student surveys and reflect a common methodological constraint in this literature rather than a systematic flaw unique to the included studies; they mean that selection bias and non-response bias cannot be excluded in any study. For descriptive purposes, the author-defined summary categories were low risk in seven studies (six scored 7 and one scored 8 out of 9 criteria) and moderate risk in two (6 and 5 out of 9); no study was rated as high risk. Because all studies share the sampling and response-rate limitations, these summary categories distinguish the studies only weakly and should not be read as evidence that the pooled estimate is free from selection bias. The full JBI appraisal results are presented in Table 2.

### 3.3. Pooled Prevalence of Anxiety—Overall Analysis

**Figure 2:**
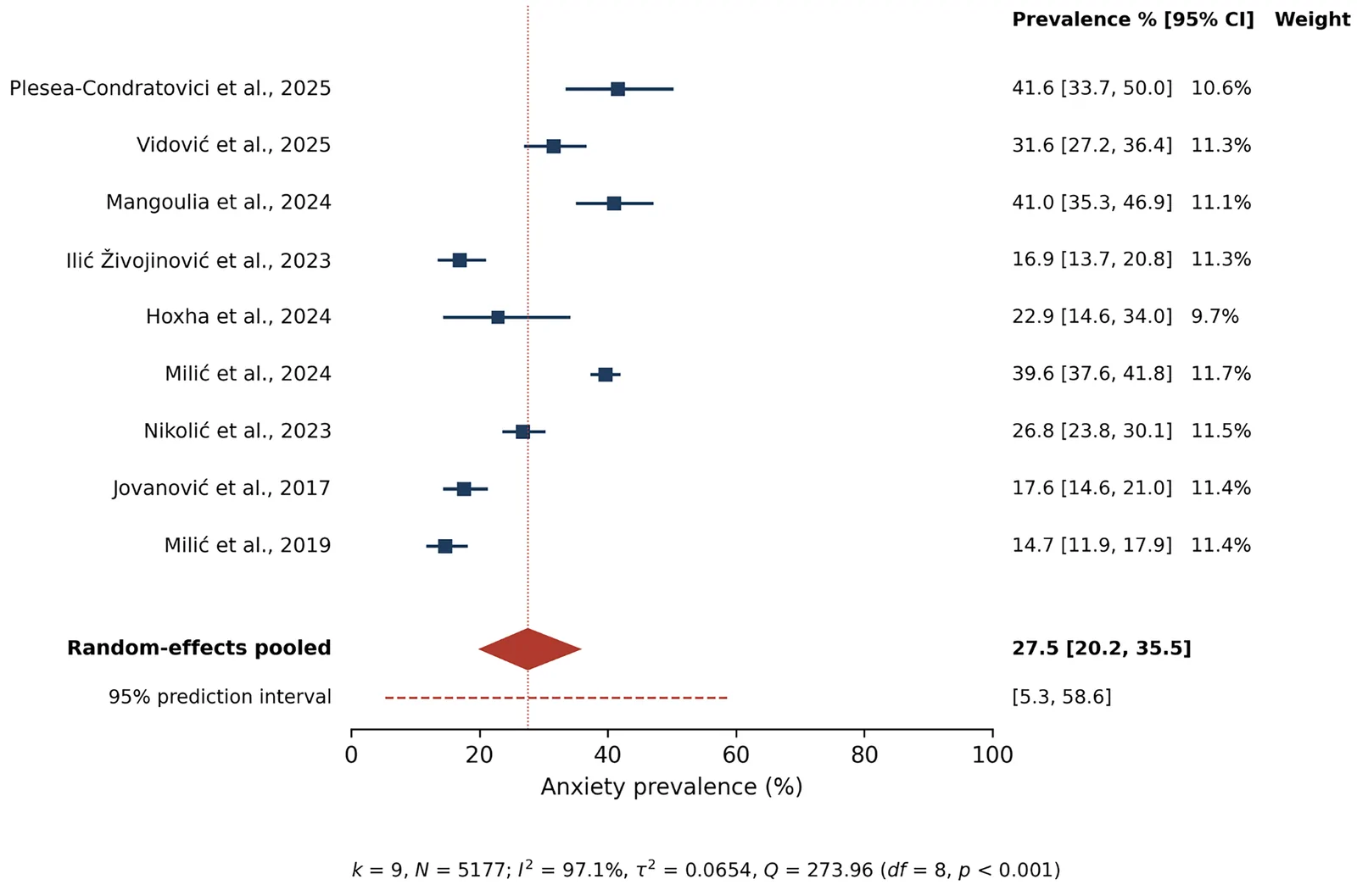
Forest plot of anxiety prevalence among medical, dental and nursing students in selected Balkan countries [31,32,33,35,43,44,45,46,47].

The random-effects meta-analysis of nine studies (total *N* = 5177), using the Freeman-Tukey double arcsine transformation and the DerSimonian-Laird estimator, yielded a pooled prevalence of anxiety of 27.5% (95% CI: 20.2–35.5%) at the screening threshold applied in each study (Table 1). Heterogeneity across studies was considerable, with an *I*^2^ of 97.1%, a Cochran’s *Q* statistic of 273.96 (*df* = 8, *p* < 0.001), and an estimated between-study variance of τ^2^ = 0.0654. The 95% prediction interval was 5.3–58.6%, indicating that the prevalence in a new study or setting could differ substantially from the pooled value. The individual study estimates, their 95% confidence intervals, and the pooled effect are illustrated in Figure 2.

### 3.4. Subgroup Analyses

#### 3.4.1. By Country

Serbia and Croatia each contributed three studies and were pooled. The pooled prevalence was 20.4% (95% CI: 14.3–27.2%) for Serbia, with considerable heterogeneity (*I*^2^ = 91.1%, *Q* = 22.55, *df* = 2, *p* < 0.001, τ^2^ = 0.0184), and 28.0% (95% CI: 13.7–45.2%) for Croatia, with extreme heterogeneity (*I*^2^ = 98.6%, *Q* = 141.26, *df* = 2, *p* < 0.001, τ^2^ = 0.0985; study estimates 14.7%, 31.6% and 39.6%). For the remaining countries, estimates are presented for individual studies without pooling: Romania 41.6% (Plesea-Condratovici et al. [47]; Greece 41.0% (Mangoulia et al. [46]); and Kosovo 22.9% (Hoxha et al. [31]). The Serbian studies ranged from 16.9% to 26.8%, whereas the Croatian studies ranged from 14.7% to 39.6%. Within each of these countries, the studies differed in population (for Serbia, medical students, and nursing and medical technician students; for Croatia, medicine and nursing students, medical students, and a mainly nursing sample), instrument and year of data collection (Serbia 2015–2022; Croatia 2018 or earlier—2025), so differences between countries cannot be attributed to country, and the Croatian pooled estimate, with its very wide confidence interval, is not informative about a Croatian prevalence.

#### 3.4.2. By Population Type

Studies of medical students only (*k* = 3) gave a pooled prevalence of 24.9% (95% CI: 17.4–33.3%; *I*^2^ = 92.5%, *Q* = 26.50, *df* = 2, *p* < 0.001, τ^2^ = 0.0245). Dental and nursing students were not pooled, as they are distinct populations; their estimates are presented individually: Hoxha et al. [31] (dental students) 22.9%, Mangoulia et al. [46] (dental and nursing students, not reported separately) 41.0%, and Jovanović et al. [43] (nursing and medical technician students) 17.6%. Two studies of mixed medicine and nursing samples were not pooled (*k* < 3): Plesea-Condratovici et al. [47] reported 41.6% and Milić et al. [33] (2019) 14.7%. Milić et al. [45] (2024; mainly nursing and other health professions), 39.6%, does not fit any of the other categories and is presented separately. These estimates are descriptive and do not support conclusions about differences between professions.

#### 3.4.3. By Assessment Instrument

Studies using the DASS-21 or DASS-42 (*k* = 5) gave a pooled prevalence of 27.8% (95% CI: 20.4–35.9%), with considerable heterogeneity (*I*^2^ = 92.8%, *Q* = 55.85, *df* = 4, *p* < 0.001, τ^2^ = 0.0349). The three studies using the GAD-7 reported 39.6% (Milić et al., 2024 [45], 16.9% (Ilić Živojinović et al. [32]) and 14.7% (Milić et al., 2019 [33]; their pooled prevalence was 23.1% (95% CI: 8.4–42.3%; *I*^2^ = 99.0%, *Q* = 197.48, *df* = 2, *p* < 0.001, τ^2^ = 0.1329), an estimate with extreme heterogeneity that is shown for completeness and should not be interpreted as a typical GAD-7 prevalence. The single study using the HADS-A reported 41.6% (Plesea-Condratovici et al. [47] with a cut-off that is not equivalent to those of the other instruments. Instrument and population are partly confounded in these data, so an instrument effect cannot be separated from differences between study populations.

#### 3.4.4. Study-Level Meta-Regression on the Proportion of Female Participants

The proportion of female participants ranged from 50.0% to 86.3% across the nine studies. In the meta-regression, each 10-percentage-point increase in the proportion of female participants was associated with an odds ratio of 1.21 for anxiety prevalence (95% CI: 0.76–1.93; *p* = 0.35), and the residual between-study variance was not reduced (τ^2^ = 0.294 with the covariate vs. 0.297 without). The association was also not significant after excluding the largest study (Milić et al., 2024 [45] OR = 1.11, 95% CI: 0.61–2.02; *p* = 0.69). With nine studies, this analysis has low power and is ecological: the result is inconclusive and it cannot show that sex does or does not influence prevalence at the individual level.

### 3.5. Sensitivity Analyses

#### 3.5.1. Exclusion of the HADS Study

Excluding Plesea-Condratovici et al. [47] the only study that used the HADS-A, gave a pooled prevalence of 26.0% (95% CI: 18.4–34.3%; *I*^2^ = 97.4%, Q = 265.17, *df* = 7, *p* < 0.001, τ^2^ = 0.0662). When the stricter HADS-A cut-off of ≥11 was applied to that study (25/137; 18.2%), the pooled prevalence of all nine studies was 25.1% (95% CI: 18.1–32.9%; *I*^2^ = 97.1%). Both results lie within the confidence interval of the primary estimate. The HADS-A cut-off therefore moves the pooled point estimate by about 1.5 to 2.5 percentage points, which is small relative to the width of the confidence interval and of the prediction interval, but these analyses do not make the HADS-A definition equivalent to the moderate-severity definitions used elsewhere.

#### 3.5.2. Exclusion of Studies by Sample Composition, Risk of Bias and Data-Collection Period

Restricting the analysis to the seven studies rated as low risk of bias (excluding Plesea-Condratovici et al. [47] and Hoxha et al. [31]) gave 26.3% (95% CI: 18.2–35.3%; *I*^2^ = 97.7%). Excluding the two studies whose data-collection period was reported only as an academic year or semester (Hoxha et al. [31] and Ilić Živojinović et al. [32]) gave 29.7% (95% CI: 21.4–38.8%; *I*^2^ = 97.4%). Excluding the largest study (Milić et al., 2024 [45] 11.7% of the random-effects weight and 39.6% of all participants; mainly nursing students) gave 26.0% (95% CI: 19.5–33.0%; *I*^2^ = 94.5%, τ^2^ = 0.0456), and excluding both studies whose samples were not restricted to medicine, dentistry or nursing programmes (Milić et al., 2024 [45] and Jovanović et al. [43]) gave 27.3% (95% CI: 20.0–35.4%; *I*^2^ = 94.6%). In all of these analyses, together with those in Section 3.5.1, the pooled estimate stayed between 25.1% and 29.7%, and heterogeneity remained very high (*I*^2^ = 94.5–97.7%).

#### 3.5.3. Leave-One-Out Analysis

In the leave-one-out analysis, pooled prevalences ranged from 26.0% (excluding Plesea-Condratovici et al. [47] Mangoulia et al. [46] or Milić et al., 2024 [45] to 29.4% (excluding Milić et al., 2019 [33], all within the 95% confidence interval of the primary estimate (20.2–35.5%). *I*^2^ ranged from 94.5% (excluding Milić et al., 2024 [45] to 97.4%, so no single study explained the heterogeneity. The results are summarised in Table 3.

#### 3.5.4. Alternative Estimators

Re-estimating the pooled prevalence with alternative methods gave similar point estimates: 27.5% (95% CI: 20.7–35.0%) with REML, 27.5% (95% CI: 20.7–34.9%) with the Paule-Mandel estimator, 27.5% (95% CI: 19.6–36.3%) with the Hartung-Knapp adjustment, 27.0% (95% CI: 20.3–34.9%) with a logit transformation, and 26.9% (95% CI: 19.9–35.1%) with a binomial-normal generalized linear mixed model. The stability of the point estimate across statistical models shows that it does not depend on the choice of model; it does not show that the underlying study populations are comparable, which is the separate question addressed by the heterogeneity statistics and the prediction interval. These analyses were not pre-specified in the protocol.

### 3.6. Risk of Publication Bias

Because fewer than ten studies were included, funnel-plot asymmetry could not be assessed reliably, and neither a funnel plot nor Egger’s test is reported. The risk of publication and availability bias could therefore not be quantified (see Section 4.4).

## 4. Discussion

### 4.1. Summary of Main Findings

This systematic review and meta-analysis is, to the best of our knowledge, the first dedicated synthesis of anxiety prevalence among medical, dental, and nursing students in selected Balkan countries. Nine cross-sectional studies published between 2017 and 2025, encompassing 5177 health science students from Serbia, Croatia, Romania, Greece and Kosovo, were included. The random-effects pooled prevalence of anxiety above the screening threshold applied in each study (moderate severity for the DASS and GAD-7; ≥8 for the HADS-A), measured with screening instruments, was 27.5% (95% CI: 20.2–35.5%). Between-study heterogeneity was very high (*I*^2^ = 97.1%) and the 95% prediction interval was wide (5.3–58.6%), so the pooled value is an average across different populations, instruments, thresholds and settings rather than a regional prevalence. The point estimate was numerically stable across statistical models and sensitivity analyses: leave-one-out estimates ranged from 26.0% to 29.4%, alternative estimators gave 26.9–27.5%, and excluding the largest study (Milić et al., 2024 [45] gave 26.0%, although heterogeneity remained very high. This stability shows that the estimate does not depend on a single study or model; it does not show that the studies sample comparable populations. Subgroup analyses were exploratory and based on few studies: the pooled estimate for medical-student samples was 24.9% (95% CI: 17.4–33.3%), whereas dental and nursing samples were not pooled and ranged from 17.6% to 41.0% across three studies; the pooled estimates were 20.4% (95% CI: 14.3–27.2%) for Serbia and 28.0% (95% CI: 13.7–45.2%) for Croatia, the latter with extreme heterogeneity (*I*^2^ = 98.6%); and a study-level meta-regression did not show a statistically significant association with the proportion of female participants, which is inconclusive given the low power. These findings do not support conclusions about differences between professions or countries.

### 4.2. Context from the International Literature

The pooled estimate of 27.5% is numerically lower than those reported by international syntheses of anxiety in medical students, although its confidence interval overlaps with most of them. Quek et al. [48], in a meta-analysis of 69 studies and over 40,000 medical students worldwide, reported a pooled anxiety prevalence of 33.8% (95% CI: 29.2–38.7%), and Ahmed et al. [14], in a meta-analysis of 83 studies of undergraduate students, reported 39.7% (95% CI: 35.7–43.6%), 37.4% (95% CI: 30.8–44.1%) in samples of medical students only, and 35.6% (95% CI: 29.5–41.8%) at a moderate-or-above threshold [14]. These comparisons are indirect and are offered as context only: those reviews differ from ours in population, time period, instruments, thresholds and methods, and therefore cannot establish whether the burden in the Balkans is higher, lower or similar to that in other regions.

For context, Lin et al. [15], in a meta-analysis of 130 studies and over 132,000 medical students, reported a pandemic-era pooled anxiety prevalence of 45% (95% CI: 40–49%). Our estimate is numerically lower, but the included studies do not form a longitudinal series and differ in data-collection period, population, instrument and threshold. This review therefore cannot test whether prevalence changed over time, and we draw no conclusion about post-pandemic normalisation.

### 4.3. Sources of Heterogeneity and Subgroup Findings

The heterogeneity observed in the primary analysis (*I*^2^ = 97.1%; prediction interval 5.3–58.6%) is very high and limits the interpretation of the pooled estimate. Several study-level characteristics may contribute, but they cannot be disentangled with nine studies, and real differences between populations and settings are equally plausible. First, the included studies used instruments with different conceptual underpinnings, item content and recommended cut-offs: the DASS-21 and DASS-42 anxiety subscales capture predominantly autonomic and somatic features of anxious arousal, the GAD-7 was developed to screen for generalised anxiety disorder in primary care, and the HADS was designed for non-psychiatric hospital patients and was applied here with a cut-off of ≥8 rather than a moderate-severity threshold, so that its operational definition of anxiety is not equivalent to that of the other instruments. Studies using the DASS (*k* = 5) yielded a pooled prevalence of 27.8% (95% CI: 20.4–35.9%) with *I*^2^ = 92.8%, so heterogeneity remained high even within one instrument family. The studies therefore combine different operational definitions of anxiety as well as different populations, and the pooled value should be read accordingly.

Second, the timing of data collection within the academic year may influence estimates. In a longitudinal study of Romanian dental students [20], which was not eligible for the present synthesis because its data collection overlapped the pandemic exclusion window, the proportion of students with anxiety was higher during the winter examination session (43.4–50.2%, depending on the university centre) than after it (28.2–37.0%). The studies included here differed in when data were collected and some did not report the timing precisely (Section 3.1), so we could not examine this source of variation. The point estimate was numerically stable in leave-one-out analyses (26.0–29.4%), but the largest study (Milić et al., 2024 [45] 2048 participants, 40% of all participants) carried 11.7% of the random-effects weight; when it was excluded, the pooled prevalence was 26.0% (95% CI: 19.5–33.0%) and *I*^2^ decreased to 94.5%, which remains very high. Heterogeneity therefore did not depend on a single study, and we cannot attribute it to a specific source. Numerical stability of the pooled value across models and exclusions should not be confused with comparability of the underlying populations: the studies differ in discipline, institution, instrument, threshold, timing and sampling, and the prediction interval shows how much the true prevalence may vary between settings. Third, differences in student discipline, institutional culture, and the proportion of female participants may contribute to between-study variation. Female sex is a consistent correlate of anxiety in health science students across international syntheses [49], and the proportion of female participants ranged from 50.0% to 86.3% across our included studies. An exploratory study-level meta-regression did not show a statistically significant association between the proportion of female participants and prevalence (odds ratio per 10-percentage-point increase 1.21, 95% CI 0.76–1.93, *p* = 0.35); this result is inconclusive rather than evidence of no association. With nine studies, however, this analysis has very low power and is subject to ecological bias, so it neither supports nor excludes an effect of sex.

### 4.4. Publication and Availability Bias

With nine studies, neither funnel-plot inspection nor Egger’s test can reliably detect or exclude small-study effects or publication bias, and we therefore draw no inference about its presence or absence. Several features of this review may increase the risk of selective availability of evidence: the restriction to English-language publications, which may have excluded studies published in regional languages, particularly from the six of the eleven countries that contributed no eligible study; the search of three bibliographic databases only; and the predominance of convenience samples. An initial restriction to open-access publications was removed after peer review and the non-open-access records were assessed under the same criteria, which added one study; this shows that the restriction would have missed eligible evidence, but it does not rule out that other eligible studies were not retrieved. The direction of any such bias cannot be determined from the present data. The pooled estimate should therefore be interpreted as applying to the published English-language literature identified here, and the true distribution across the region may differ.

### 4.5. Implications and Need for Further Data

This review synthesises cross-sectional prevalence studies and did not evaluate any intervention; the points below are therefore implications for further work rather than evidence-based recommendations. The pooled estimate (27.5%) and, above all, the wide range between individual studies (14.7% to 41.6%) indicate that a substantial proportion of the health science students in the studied samples reported anxiety symptoms above the screening thresholds applied, although this cannot be generalised to the whole region or to every institution (Section 4.4). These findings are compatible with a case for institutions to collect their own data using validated instruments and to report the threshold applied and the timing of data collection, so that local needs can be assessed. Whether routine screening or specific support measures, such as peer support, counselling services or curricular changes, would reduce anxiety among Balkan students was not assessed by any included study. Their effectiveness has to be judged from evidence outside this review, including studies of such interventions in other settings [50], and we therefore do not recommend specific programmes on the basis of the present results.

### 4.6. Strengths and Limitations

Several strengths of this review merit acknowledgement. To the best of our knowledge, it is the first dedicated synthesis of anxiety prevalence among medical, dental, and nursing students in selected Balkan countries, addressing a regional evidence gap. The review was prospectively registered in PROSPERO, followed the PRISMA 2020 guidelines [30], and reports its deviations from the registered protocol transparently (Section 2.7). Screening, data extraction, and quality appraisal with the JBI Critical Appraisal Checklist for Prevalence Studies [36] were performed independently by two reviewers, with complete agreement (Cohen’s κ = 1.00). Only studies using a validated instrument with a pre-defined cut-off were pooled; eight of the nine used a moderate-severity threshold, whereas the HADS-A study used a lower cut-off (≥8) that is not equivalent, and this difference was examined in sensitivity analyses (Section 3.5.1). The primary random-effects analysis was complemented by prediction intervals, leave-one-out analyses, and alternative estimators, which gave consistent point estimates.

Several limitations also warrant explicit discussion. First, the number of eligible studies (*k* = 9) is small relative to conventional meta-analytic standards and limits the precision of all pooled estimates, the statistical power of bias-detection methods, and the power of subgroup analyses and meta-regression.

Second, the restriction to English-language publications—defined at the outset of the searches (Section 2.2)—may have excluded relevant studies published in regional languages, particularly in countries such as Bulgaria, Slovenia, Albania, North Macedonia, Bosnia and Herzegovina, and Montenegro, which contributed no eligible studies to the final synthesis. An initial restriction to open-access publications was removed after peer review because open-access status is unrelated to study quality or population; the searches were repeated and the non-open-access records were assessed under the same criteria (Section 2.1 and Section 2.7). Because this change was made after the results of the earlier analyses were known, it is reported as a post hoc deviation from the registered protocol, although it followed the reviewers’ comments and not the prevalence estimates. Third, the included studies relied uniformly on self-report screening instruments, which identify probable cases rather than diagnostically confirmed anxiety disorders and may overestimate the prevalence of clinically meaningful pathology.

Fourth, all included studies used convenience sampling—reflected in Q2 of the JBI checklist not being met in any study and Q9 in seven of the nine—which introduces the possibility of self-selection bias, with anxious students potentially being either more or less likely to participate in mental health surveys. Even where a response rate was reported (72.7% in Milić et al., 2019 [33], non-respondents may differ from respondents.

Fifth, the between-study heterogeneity was very high (*I*^2^ = 97.1%) and persisted across all subgroup and sensitivity analyses. The random-effects model with the Freeman–Tukey transformation and the DerSimonian–Laird estimator may yield too narrow confidence intervals when few studies are pooled and between-study variance is large. Alternative estimators and the Hartung–Knapp adjustment produced similar point estimates (26.9–27.5%) and somewhat wider intervals (up to 19.6–36.3% with the Hartung–Knapp adjustment), but this similarity concerns the statistical model, not the populations: the prediction interval (5.3–58.6%) shows that the true prevalence in a given setting may differ substantially. The pooled value should therefore be interpreted as an average across heterogeneous studies, not as a regional prevalence or benchmark. Sixth, the evidence base is uneven. Nine studies from five of the eleven countries contributed data (Serbia three, Croatia three, and Romania, Greece and Kosovo one each); medicine-only samples were available in three studies and dentistry-only in one; and the largest study (Milić et al., 2024 [45] 11.7% of the random-effects weight and 40% of all participants) consisted mainly of nursing students (69.2%) together with students of other health professions. The pooled estimate therefore cannot be attributed to any single discipline or country, and comparisons between professions or countries are not supported by these data. Excluding the two studies whose samples were not restricted to medicine, dentistry or nursing gave a similar estimate (27.3%, 95% CI: 20.0–35.4%; *k* = 7). Seventh, the operational definitions of anxiety are not equivalent across instruments: eight studies used a moderate-severity cut-off, whereas the HADS-A study used a cut-off of ≥8 that identifies borderline as well as abnormal scores. Sensitivity analyses (Section 3.5.1) show that this study changes the pooled point estimate only modestly, but they cannot make the definitions equivalent, and the DASS, GAD-7 and HADS-A also capture different aspects of anxiety (Section 4.3).

### 4.7. Future Research Directions

Several priorities for future research in this region can be identified on the basis of the present synthesis. First, primary prevalence studies are needed from the Balkan countries for which no eligible English-language study was identified in the present review, despite their inclusion in the search and eligibility criteria, including Bulgaria, Slovenia, Albania, North Macedonia, Bosnia and Herzegovina, and Montenegro, ideally using a harmonised methodological framework that specifies the assessment instrument, the cut-off applied, the timing within the academic calendar, and the recruitment strategy. Second, longitudinal designs that follow cohorts of health science students across the duration of their training would substantially improve causal inference regarding the predictors and consequences of anxiety, addressing the limitation identified in the present synthesis whereby cross-sectional designs cannot disentangle anxiety from its temporal context. Third, future studies should report disaggregated estimates by sex, year of study, and discipline to permit more granular subgroup analysis at the regional level. Fourth, intervention research evaluating context-appropriate, scalable, and culturally sensitive mental health programmes in Balkan medical schools—including digital cognitive-behavioural interventions, mindfulness-based programmes, and structured peer-support models—represents a logical next step beyond descriptive epidemiology [51]. Finally, institutions and national bodies should collect local data using harmonised instruments and thresholds and report the timing of data collection, so that future syntheses can pool comparable estimates. Given the very high heterogeneity observed, the present pooled estimate should not be used as a reference value against which individual institutions are judged.

## 5. Conclusions

This systematic review and meta-analysis of nine cross-sectional studies (5177 students) from five Balkan countries (Croatia, Romania, Greece, Kosovo and Serbia) found a pooled prevalence of anxiety above the screening threshold applied in each study (moderate severity for the DASS and GAD-7; ≥8 for the HADS-A) of 27.5% (95% CI: 20.2–35.5%), with very high between-study heterogeneity (*I*^2^ = 97.1%; prediction interval 5.3–58.6%). This value is an average across studies that differ in population, instrument, threshold and timing; it should not be read as a regional prevalence or benchmark, and the sources of the heterogeneity could not be determined. It does not represent any single discipline or country and should not be used to compare professions or countries. The findings apply to the published English-language literature identified, and six of the eleven countries searched contributed no eligible study. The design of this review does not allow conclusions about how prevalence compares with other regions or has changed over time, nor about the effectiveness of any intervention. A considerable proportion of the students in the included samples reported anxiety symptoms, which supports the need for further primary research in the region using harmonised instruments and thresholds, reporting of the timing of data collection, and more representative sampling. These findings should be regarded as preliminary.

## Figures and Tables

**Table 1:** Characteristics of Included Studies.

Study	Country	Population	Design	Instrument *	*N*	Prevalence (%)	Female (%)
Plesea-Condratovici et al., 2025 [47]	Romania	Medicine + Nursing	Cross-sectional	HADS-A	137	41.6	77.4
Vidović et al., 2025 [35]	Croatia	Medicine	Cross-sectional	DASS-21	386	31.6	75.1
Mangoulia et al., 2024 [46]	Greece	Dentistry + Nursing	Cross-sectional	DASS-21	271	41	68.6
Ilić Živojinović et al., 2023 [32]	Serbia	Medicine	Cross-sectional	GAD-7	431	16.9	65
Hoxha et al., 2024 [31]	Kosovo	Dentistry	Cross-sectional	DASS-21	70	22.9	50.0
Milić et al., 2024 [45] †	Croatia	Health sciences: nursing, physiotherapy, other health professions	Cross-sectional	GAD-7	2048	39.6	86.3
Nikolić et al., 2023 [44]	Serbia	Medicine	Cross-sectional	DASS-21	761	26.8	68.5
Jovanović et al., 2017 [43]	Serbia	Nursing/medical technician	Cross-sectional	DASS-42	535	17.6	80.7
Milić et al., 2019 [33] †	Croatia	Medicine + Nursing	Cross-sectional	GAD-7	538	14.7	69.2

DASS-21 = Depression Anxiety Stress Scales—21 items; DASS-42 = Depression Anxiety Stress Scales—42 items; GAD-7 = Generalized Anxiety Disorder 7-item scale; HADS = Hospital Anxiety and Depression Scale. * Cut-off: DASS-21 and DASS-42 anxiety subscale score ≥ 10 (moderate or above); GAD-7 score ≥ 10; HADS-A score ≥ 8 (borderline abnormal or above). † Prevalence for Milić et al. [45] is based on 2048 participants with GAD-7 data and for Milić et al. 2019 [33] on 538 of 562 participants with GAD-7 data (79 cases); the proportion of female participants refers to all participants (2137 and 562, respectively).

**Table 2:** JBI Critical Appraisal Checklist for Prevalence Studies.

Study	Q1	Q2	Q3	Q4	Q5	Q6	Q7	Q8	Q9	Total (/9)	Risk of Bias
Plesea-Condratovici et al., 2025 [47]	✓	X	X	✓	✓	✓	✓	✓	X	6	Moderate
Vidović et al., 2025 [35]	✓	X	✓	✓	✓	✓	✓	✓	X	7	Low
Mangoulia et al., 2024 [46]	✓	X	✓	✓	✓	✓	✓	✓	X	7	Low
Ilić Živojinović et al., 2023 [32]	X	X	✓	✓	✓	✓	✓	✓	✓	7	Low
Hoxha et al., 2024 [31]	X	X	X	✓	✓	✓	✓	✓	X	5	Moderate
Milić et al., 2024 [45]	✓	X	✓	✓	✓	✓	✓	✓	X	7	Low
Nikolić et al., 2023 [44]	✓	X	✓	✓	✓	✓	✓	✓	X	7	Low
Jovanović et al., 2017 [43]	✓	X	✓	✓	✓	✓	✓	✓	X	7	Low
Milić et al., 2019 [33]	✓	X	✓	✓	✓	✓	✓	✓	✓	8	Low

Q1 = Was the sample frame appropriate for the target population? Q2 = Were participants recruited in an appropriate way? Q3 = Was the sample size adequate? Q4 = Were study subjects and setting described in detail? Q5 = Was data analysis conducted with sufficient coverage of the sample? Q6 = Were valid methods used for identification of the condition? Q7 = Was the condition measured in a standard and reliable way for all participants? Q8 = Was appropriate statistical analysis used? Q9 = Was the response rate adequate, and if not, was the low response rate managed appropriately? ✓ = Yes; X = No. Risk of Bias: Low = ≥7 criteria met; Moderate = 4–6, High ≤ 3 criteria met.

**Table 3:** Leave-One-Out Sensitivity Analysis.

Study Excluded	*k*	Pooled Prevalence (%)	95% CI	*I*^2^ (%)
Plesea-Condratovici et al. 2025 [47]	8	26.0	18.4–34.3%	97.4
Vidović et al., 2025 [35]	8	27.0	18.9–36.0%	97.4
Mangoulia et al., 2024 [46]	8	26.0	18.3–34.4%	97.3
Ilić Živojinović et al., 2023 [32]	8	29.0	21.3–37.4%	97.0
Hoxha et al., 2024 [31]	8	28.0	20.2–36.5%	97.4
Milić et al., 2024 [45]	8	26.0	19.5–33.0%	94.5
Nikolić et al., 2023 [44]	8	27.6	19.1–37.1%	97.4
Jovanović et al., 2017 [43]	8	28.9	21.2–37.3%	96.9
Milić et al., 2019 [33]	8	29.4	22.1–37.2%	96.4
Overall (all studies)	9	27.5	20.2–35.5%	97.1

## Data Availability

The data analysed in this study were extracted from the published articles listed in Table 1 and the reference list. The extracted data are provided in Table 1 and Supplementary Tables S1–S3. No new primary data were generated.

## Supplementary Materials

The following supporting information can be downloaded at: https://www.mdpi.com/article/10.3390/jcm15218566/s1, Table S1 (database searches: final search, earlier open-access runs and novelty check), Table S2 (full-text exclusions), Table S3 (reports from the same population) and Table S4 (PRISMA 2020 checklist).

## Abbreviations

The following abbreviations are used in this manuscript:

GAD-7	Generalized Anxiety Disorder 7-item scale
BAI	Beck Anxiety Inventory
DASS-21	Depression, Anxiety and Stress Scale, 21-item version
HADS	Hospital Anxiety and Depression Scale
BMI	Body Mass Index
PRISMA	Preferred Reporting Items for Systematic Reviews and Meta-Analyses
PROSPERO	International Prospective Register of Systematic Reviews
JBI	Joanna Briggs Institute

## Appendix A

**Table A1:** Search strategy for each database.

Source	Search Strategy	Articles Retrieved
PubMed	(“Students, Medical” OR “medical student*”[tiab] OR “medical undergraduate*”[tiab] OR “Students, Nursing” OR “nursing student*”[tiab] OR “Students, Dental” OR “dental student*”[tiab] OR “dentistry student*”[tiab] OR “health science* student*”[tiab]) AND (“Anxiety” OR “Anxiety Disorders” OR “anxiety”[tiab] OR “anxious”[tiab]) AND (“Albania” OR “Bosnia and Herzegovina” OR “Bulgaria” OR “Croatia” OR “Greece” OR “Montenegro” OR “Republic of North Macedonia” OR “Romania” OR “Serbia” OR “Slovenia” OR “Albania”[tiab] OR “Bosnia”[tiab] OR “Herzegovina”[tiab] OR “Bulgaria”[tiab] OR “Croatia”[tiab] OR “Greece”[tiab] OR “Kosovo”[tiab] OR “Montenegro”[tiab] OR “North Macedonia”[tiab] OR “Macedonia”[tiab] OR “Romania”[tiab] OR “Serbia”[tiab] OR “Slovenia”[tiab]) Filters:English	101
Web of Science	TS=(“medical student*” OR “medical undergraduate*” OR “nursing student*” OR “dental student*” OR “dentistry student*” OR “health science* student*”) AND TS=(“anxiety” OR “anxiety disorder*” OR “anxious”) AND TS=(“Albania” OR “Bosnia” OR “Herzegovina” OR “Bulgaria” OR “Croatia” OR “Greece” OR “Kosovo” OR “Montenegro” OR “North Macedonia” OR “Macedonia” OR “Romania” OR “Serbia” OR “Slovenia”) Filters:English	84
Scopus	TITLE-ABS-KEY (“medical student*” OR “medical undergraduate*” OR “nursing student*” OR “dental student*” OR “dentistry student*” OR “health science* student*”) AND TITLE-ABS-KEY (“anxiety” OR “anxiety disorder*” OR “anxious”) AND TITLE-ABS-KEY (“Albania” OR “Bosnia” OR “Herzegovina” OR “Bulgaria” OR “Croatia” OR “Greece” OR “Kosovo” OR “Montenegro” OR “North Macedonia” OR “Macedonia” OR “Romania” OR “Serbia” OR “Slovenia”) Filters:English	96

Searches were first run on 31 March 2026 and re-run on 29 September 2026 with an open-access filter; after peer review they were repeated on 12 October 2026 without the open-access filter. The table reports the final run (total 281 records). Records published after 31 March 2026 were removed manually after export.

## References

[1] Zhou J., Li S., Song Y., Ying J., Luo Z., Shan S., Zhou L., Zha J., Wang X., Song P. (2025). Global, Regional, and National Trends in the Burden of Anxiety Disorders From 1992 to 2021: An Age–Period–Cohort Analysis Based on the Global Burden of Disease Study 2021. Depress. Anxiety.

[2] World Health Organization (2022). World Mental Health Report: Transforming Mental Health for All.

[3] Zhang Z., Chen X., Wu S., Chen X., Wang X., Liu C., Zeng N., Liu Y., Huo T., Liu X. (2026). Global, Regional and National Burden of Anxiety and Depression Disorders from 1990 to 2021, and Forecasts up to 2040. J. Affect. Disord..

[4] Barbui C., Alonso J., Chisholm D., Evans-Lacko S., Keynejad R.C., Lazeri L., Miah N., Valuckiene Z., Gastaldon C. (2025). Mental Health Service Coverage and Gaps among Adults in Europe: A Systematic Review. Lancet Reg. Health—Eur..

[5] Hook K., Bogdanov S. (2021). Mental Health Care in Eastern Europe and Central Asia: An Analysis of Needs and a Call for Greater Investment. Lancet Reg. Health—Eur..

[6] Dionisie V., Puiu M.G., Manea M.C., Moisa E., Dumitru A.M., Ibadula L., Mares A.M., Varlam C.I., Manea M. (2025). Factors Associated with the Revolving Door Phenomenon in Patients with Schizophrenia: Results from an Acute Psychiatric Hospital in Romania. Front. Psychiatry.

[7] Spitzer R.L., Kroenke K., Williams J.B.W., Löwe B. (2006). A Brief Measure for Assessing Generalized Anxiety Disorder: The GAD-7. Arch. Intern. Med..

[8] Henry J.D., Crawford J.R. (2005). The Short-Form Version of the Depression Anxiety Stress Scales (DASS-21): Construct Validity and Normative Data in a Large Non-Clinical Sample. Br. J. Clin. Psychol..

[9] Zigmond A.S., Snaith R.P. (1983). The Hospital Anxiety and Depression Scale. Acta Psychiatr. Scand..

[10] Plummer F., Manea L., Trepel D., McMillan D. (2016). Screening for Anxiety Disorders with the GAD-7 and GAD-2: A Systematic Review and Diagnostic Metaanalysis. Gen. Hosp. Psychiatry.

[11] Lai A.Y.-K., Yeung W.-F. (2022). Editorial: Mental Health of Higher Education Students. Front. Psychiatry.

[12] Bloomfield L.S.P., Fudolig M.I., Kim J.N., Llorin J., Lovato J., McGinnis E.W., McGinnis R.S., Price M., Ricketts T.H., Sheridan Dodds P. (2025). Predictors of Anxiety Trajectories in Cohort of First-Year College Students. JAACAP Open.

[13] Li W., Zhao Z., Chen D., Peng Y., Lu Z. (2022). Prevalence and Associated Factors of Depression and Anxiety Symptoms among College Students: A Systematic Review and Meta-analysis. Child Psychol. Psychiatry.

[14] Ahmed I., Hazell C.M., Edwards B., Glazebrook C., Davies E.B. (2023). A Systematic Review and Meta-Analysis of Studies Exploring Prevalence of Non-Specific Anxiety in Undergraduate University Students. BMC Psychiatry.

[15] Lin Y.-K., Saragih I.D., Lin C.-J., Liu H.-L., Chen C.-W., Yeh Y.-S. (2024). Global Prevalence of Anxiety and Depression among Medical Students during the COVID-19 Pandemic: A Systematic Review and Meta-Analysis. BMC Psychol..

[16] Kiss H., Pikó B.F. (2025). Risk and Protective Factors of Student Burnout among Medical Students: A Multivariate Analysis. BMC Med. Educ..

[17] Hassan S.M., Alqahtani N.M., Alshahrani S.M., Abuelnasr A.M., Alhefzy A.A., Al Assiri K.W., Al Assiri B.W., Alharthi O.S., Alharbi M.M. (2026). Mental Health Burden and Service Barriers among Medical and Health Sciences Students: Prevalence and Determinants from a Cross-Sectional Analysis. Front. Psychol..

[18] García-Rivas A., Martos-Cabrera M.B., Membrive Jiménez M.J., Aguayo-Estremera R., Suleiman Martos N., Albendín-García L., Gómez-Urquiza J.L. (2024). Anxiety in Nursing Students during the COVID-19 Pandemic: Systematic Review and Meta-Analysis. Healthcare.

[19] Vo T.N., Chiu H.-Y., Chuang Y.-H., Huang H.-C. (2023). Prevalence of Stress and Anxiety Among Nursing Students: A Systematic Review and Meta-Analysis. Nurse Educ..

[20] Rotaru D.I., Chisnoiu R.M., Bolboacă S.D., Gileru E.A., Chisnoiu A.M., Delean A.G. (2024). Insights into Self-Evaluated Stress, Anxiety, and Depression among Dental Students. Sci. Rep..

[21] Santabarbara J., Idoiaga N., Ozamiz-Etxebarria N., Bueno-Notivol J. (2021). Prevalence of Anxiety in Dental Students during the COVID-19 Outbreak: A Meta-Analysis. Int. J. Environ. Res. Public Health.

[22] Higgins J.P.T., Thomas J., Chandler J., Cumpston M., Li T., Page M.J., Welch V.A. (2019). Cochrane Handbook for Systematic Reviews of Interventions.

[23] Sinval J., Oliveira P., Novais F., Almeida C.M., Telles-Correia D. (2025). Exploring the Impact of Depression, Anxiety, Stress, Academic Engagement, and Dropout Intention on Medical Students’ Academic Performance: A Prospective Study. J. Affect. Disord..

[24] Cruz A.F.P., Pereira W.D.S., Ribeiro N.G., Sousa J.N., Macedo M.T.S., Baldo T.D.O.F., Baldo M.P. (2026). Anxiety in the Academic Environment: Impacts on Quality of Life and Well-Being of University Students. Int. J. Educ. Res..

[25] Turki M., Abid W., Khardani O., Halouani N., Bakallah M., Ellouze S., Aloulou J. (2024). Risk Factors for Suicidal Ideations and Suicide Attempts among Medical Students. Eur. Psychiatr..

[26] Hyseni Duraku Z., Davis H., Blakaj A., Ahmedi Seferi A., Mullaj K., Greiçevci V. (2024). Mental Health Awareness, Stigma, and Help-Seeking Attitudes among Albanian University Students in the Western Balkans: A Qualitative Study. Front. Public Health.

[27] Zhang S.X., Miller S.O., Xu W., Yin A., Chen B.Z., Delios A., Dong R.K., Chen R.Z., McIntyre R.S., Wan X. (2022). Meta-Analytic Evidence of Depression and Anxiety in Eastern Europe during the COVID-19 Pandemic. Eur. J. Psychotraumatol..

[28] Vidović S., Kotromanović S., Pogorelić Z. (2024). Depression, Anxiety, and Stress Symptoms Among Students in Croatia During the COVID-19 Pandemic: A Systematic Review. J. Clin. Med..

[29] Winkler P., Krupchanka D., Roberts T., Kondratova L., Machů V., Höschl C., Sartorius N., Van Voren R., Aizberg O., Bitter I. (2017). A Blind Spot on the Global Mental Health Map: A Scoping Review of 25 Years’ Development of Mental Health Care for People with Severe Mental Illnesses in Central and Eastern Europe. Lancet Psychiatry.

[30] Page M.J., McKenzie J.E., Bossuyt P.M., Boutron I., Hoffmann T.C., Mulrow C.D., Shamseer L., Tetzlaff J.M., Akl E.A., Brennan S.E. (2021). The PRISMA 2020 Statement: An Updated Guideline for Reporting Systematic Reviews. BMJ.

[31] Hoxha V., Cakolli V., Bajgora V. (2024). Levels of Stress, Anxiety and Depression among Students at Alma Mater Europaea Campus College “Rezonanca,” Kosovo. Int. J. Biomed..

[32] Ilić Živojinović J., Soldatović I., Backović D., Vukašinović D., Babić S., Tomanić M., Ilić B., Vlaisavljević Ž. (2023). Personal Listening Device Use and Attitude to Noise in Relation to Depression and Anxiety among Medical Students. Noise Health.

[33] Milić J., Škrlec I., Milić Vranješ I., Podgornjak M., Heffer M. (2019). High Levels of Depression and Anxiety among Croatian Medical and Nursing Students and the Correlation between Subjective Happiness and Personality Traits. Int. Rev. Psychiatry.

[34] Ouzzani M., Hammady H., Fedorowicz Z., Elmagarmid A. (2016). Rayyan—A Web and Mobile App for Systematic Reviews. Syst. Rev..

[35] Vidović S., Rakić N., Kraštek S., Pešikan A., Degmečić D., Zibar L., Labak I., Heffer M., Pogorelić Z. (2025). Sleep Quality and Mental Health Among Medical Students: A Cross-Sectional Study. J. Clin. Med..

[36] Aromataris E., Lockwood C., Porritt K., Pilla B., Jordan Z. (2024). JBI Manual for Evidence Synthesis.

[37] Harris C.R., Millman K.J., Van Der Walt S.J., Gommers R., Virtanen P., Cournapeau D., Wieser E., Taylor J., Berg S., Smith N.J. (2020). Array Programming with NumPy. Nature.

[38] Virtanen P., Gommers R., Oliphant T.E., Haberland M., Reddy T., Cournapeau D., Burovski E., Peterson P., Weckesser W., Bright J. (2020). SciPy 1.0: Fundamental Algorithms for Scientific Computing in Python. Nat. Methods.

[39] Freeman M.F., Tukey J.W. (1950). Transformations Related to the Angular and the Square Root. Ann. Math. Stat..

[40] DerSimonian R., Laird N. (1986). Meta-Analysis in Clinical Trials. Control. Clin. Trials.

[41] Egger M., Smith G.D., Schneider M., Minder C. (1997). Bias in Meta-Analysis Detected by a Simple, Graphical Test. BMJ.

[42] Sterne J.A.C., Sutton A.J., Ioannidis J.P.A., Terrin N., Jones D.R., Lau J., Carpenter J., Rucker G., Harbord R.M., Schmid C.H. (2011). Recommendations for Examining and Interpreting Funnel Plot Asymmetry in Meta-Analyses of Randomised Controlled Trials. BMJ.

[43] Jovanović V.R., Đonović N., Dutina A. (2017). Factors Associated with the Depression, Anxiety and Stress among High Medical School of Professional Studies Students. Med. Čas..

[44] Nikolić A., Bukurov B., Kocić I., Vuković M., Ladjević N., Vrhovac M., Pavlović Z., Grujičić J., Kisić D., Sipetić S. (2023). Smartphone Addiction, Sleep Quality, Depression, Anxiety, and Stress among Medical Students. Front. Public Health.

[45] Milić J., Skitarelić N., Majstorović D., Zoranić S., Čivljak M., Ivanišević K., Marendić M., Mesarić J., Puharić Z., Neuberg M. (2024). Levels of Depression, Anxiety and Subjective Happiness among Health Sciences Students in Croatia: A Multi-Centric Cross-Sectional Study. BMC Psychiatry.

[46] Mangoulia P., Kanellopoulou A., Manta G., Chrysochoou G., Dimitriou E., Kalogerakou T., Antoniadou M. (2024). Exploring the Levels of Stress, Anxiety, Depression, Resilience, Hope, and Spiritual Well-Being Among Greek Dentistry and Nursing Students in Response to Academic Responsibilities Two Years After the COVID-19 Pandemic. Healthcare.

[47] Pleșea-Condratovici C., Dionisie V., Moroianu L.-A., Serban P.-S., Plesea-Condratovici V., Arbune M. (2025). Perception Versus Actual Weight: Body Image Dissatisfaction as a Stronger Correlate of Anxiety and Depression than BMI Among Romanian Health Sciences Students. Healthcare.

[48] Tian-Ci Quek T., Wai-San Tam W., Tran B.X., Zhang M., Zhang Z., Su-Hui Ho C., Chun-Man Ho R. (2019). The Global Prevalence of Anxiety Among Medical Students: A Meta-Analysis. Int. J. Environ. Res. Public Health.

[49] Bandelow B., Michaelis S. (2015). Epidemiology of Anxiety Disorders in the 21st Century. Dialogues Clin. Neurosci..

[50] Dyrbye L.N., Shanafelt T.D., Sinsky C.A., Cipriano P.F., Bhatt J., Ommaya A., West C.P., Meyers D. (2017). Burnout Among Health Care Professionals: A Call to Explore and Address This Underrecognized Threat to Safe, High-Quality Care. NAM Perspect..

[51] Harrer M., Adam S.H., Baumeister H., Cuijpers P., Karyotaki E., Auerbach R.P., Kessler R.C., Bruffaerts R., Berking M., Ebert D.D. (2019). Internet Interventions for Mental Health in University Students: A Systematic Review and Meta-analysis. Int. J. Methods Psych. Res..

